# Antiulcerogenic Potential of the Ethanolic Extract of *Ceiba speciosa* (A. St.-Hil.) Ravenna Evaluated by In Vitro and In Vivo Studies

**DOI:** 10.3390/ijms232415634

**Published:** 2022-12-09

**Authors:** Juliana Andréa Dörr, Fernanda Majolo, Luísa Bortoluzzi, Evelin Zen de Vargas, Joana Silva, Manoela Pasini, Stefani Natali Stoll, Rafael Lopes da Rosa, Mariana Moreira Figueira, Márcio Fronza, Walter O. Beys-da-Silva, Alice Martins, Helena Gaspar, Rui P. Pedrosa, Stefan Laufer, Márcia Inês Goettert

**Affiliations:** 1 Graduate Program in Biotechnology (PPGBiotec), University of Vale do Taquari-Univates, Av. Avelino Talini, 171, Lajeado 95914-014, RS, Brazil; 2 Graduate Program in Medicine Sciences (PPGCM), University of Vale do Taquari-Univates, Av. Avelino Talini, 171, Lajeado 95914-014, RS, Brazil; 3 MARE—Marine and Environmental Sciences Centre/ARNET—Aquatic Research Network, Polytechnic of Leiria, 2520-630 Peniche, Portugal; 4 Institute of Pharmacy, Universidade Federal do Rio Grande do Sul, Porto Alegre 91501-970, RS, Brazil; 5 Graduate Program in Pharmaceutical Sciences, Natural Products Laboratory, Vila Velha University, Vila Velha 29102-920, ES, Brazil; 6 BioISI—Biosystems and Integrative Sciences Institute, Faculty of Sciences, University of Lisbon, 1749-016 Lisboa, Portugal; 7 Department of Pharmaceutical and Medicinal Chemistry, Institute of Pharmacy, Eberhard Karls Universität Tübingen, D 72076 Tuebingen, Germany; 8 Tuübingen Center for Academic Drug Discovery-TÜCADD, Eberhard Karls Universität Tübingen, D 72076 Tübingen, Germany; 9 Cluster of Excellence iFIT (EXC 2180) ‘Image-Guided and Functionally Instructed Tumor Therapies’, Eberhard Karls Universität Tübingen, D 72076 Tübingen, Germany

**Keywords:** antioxidant activity, anti-inflammatory, *Ceiba* genus, gastrointestinal diseases, inflammation, peptic ulcer

## Abstract

Gastrointestinal diseases, such as peptic ulcers, are caused by a damage in the gastric mucosa provoked by several factors. This stomach injury is regulated by many inflammatory mediators and is commonly treated with proton-pump inhibitors, histamine H2 receptor blockers and antacids. However, various medicinal plants have demonstrated positive effects on gastric ulcer treatment, including plants of the *Ceiba* genus. The aim of this study was to evaluate the antiulcer and anti-inflammatory activities of the stem bark ethanolic extract of *Ceiba speciosa* (A. St.-Hil.) Ravenna. We performed a preliminary quantification of phenolic compounds by high-performance liquid chromatography-diode array detection (HPLC-DAD), followed by the prospection of other chemical groups through nuclear magnetic resonance (NMR) spectroscopy. A set of in vitro assays was used to evaluate the extract potential regarding its antioxidant activity (DPPH: 19.83 ± 0.34 µg/mL; TPC: 307.20 ± 6.20 mg GAE/g of extract), effects on cell viability and on the release of TNF-α in whole human blood. Additionally, in vivo assays were performed to evaluate the leukocyte accumulation and total protein quantification in carrageenan-induced air pouch, as well as the antiulcerogenic effect of the extract on an ethanol-induced ulcer in rats. The extract contains flavonoids and phenolic compounds, as well as sugars and quinic acid derivatives exhibiting potent antioxidant activity and low toxicity. The extract reduced the release of TNF-α in human blood and inhibited the activity of p38α (1.66 µg/mL), JAK3 (5.25 µg/mL), and JNK3 (8.34 µg/mL). Moreover, it reduced the leukocyte recruitment on the pouch exudate and the formation of edema, reverting the effects caused by carrageenan. The extract presented a significant prevention of ulcer formation and a higher reduction than the reference drug, Omeprazole. Therefore, *C. speciosa* extract has demonstrated relevant therapeutic potential for the treatment of gastric diseases, deserving the continuation of further studies to unveil the mechanisms of action of plant bioactive ingredients.

## 1. Introduction

Peptic ulcer is a chronic disease of the gastrointestinal tract that is usually located in the stomach or proximal duodenum [1,2]. Peptic ulcer causes a gastric mucosal damage, and the main causes are infection by *Helicobacter pylori*, stress, cigarette smoking, alcohol consumption, the administration of steroidal and nonsteroidal anti-inflammatory drugs (NSAIDs), nutritional deficiencies, and age-related decline in prostaglandin levels [3,4]. In the stomach, prostaglandins stimulate mucus and bicarbonate secretion and increase mucosal blood flow, improving the resistance of the gastric mucosa to injury [5]. NSAIDs are used to treat pain and inflammation and are involved in gastric mucosal damage, through the inhibition of cyclooxygenase-1 (COX-1), which is responsible for prostaglandins synthesis [1,2,3]. Consequently, the loss of prostaglandins is associated with low mucus and bicarbonate secretion, decreased mucosal blood flow, and inhibition of cell proliferation [1]. When mucosal integrity is challenged, cyclooxygenase-2 (COX-2) can produce prostaglandins for the maintenance of blood flow. Prostaglandins regulate several inflammatory mediators, such as tumor necrosis factor-α (TNF-α), that can play a role in mucosal injury in certain circumstances [5]. Inflammatory response is a basic protective immune process of the organism to protect the body from various injurious stimuli and it is known that the p38 mitogen-activated protein kinase (MAPK) is involved in the production of inflammatory mediators, including TNF-α and COX-2, which are important mediators in stomach inflammation [5,6]. In the same context, in macrophages, the prostaglandin 2 (PGE_2_)-mediated activation of p38 MAPK suppressed the activation of the c-Jun N-terminal kinases (JNK) pathway and it was reported that PGE2 inhibited the factor nuclear *kappa* B (NF-κB) activity [7]. Inhibition of gastric acid secretion by proton-pump inhibitors (PPIs), histamine H2 receptor blockers, eradication of *H. pylori* and antacids are the typical treatments for gastric ulcer [3,8,9]. Nevertheless, they have some adverse effects, such as gynaecomastia in men and galactorrhoea in women, nausea, abdominal pain, constipation, osteoporotic bone fracture, and deficiencies of iron and magnesium [3,9]. Due to these side effects, more research of new and safe drugs with gastroprotective activity is needed.

Natural products derived from plants have a significant role in drug discovery, offering a better protection for individuals [9,10,11], and various medicinal plants are known for their effectiveness in gastric ulcer treatment [12]. Antioxidant, cytoprotective, and anti-secretory action are the properties usually described for plants with antiulcer activity [12]. Plants of the *Ceiba* genus (Malvaceae family) have shown pharmacological potential as antioxidant [13,14,15], antiulcerogenic [9,13], and hypoglycemic [16] agents. A methanolic extract of *Ceiba pentandra* stem bark inhibited the gastric ulcer induced in rats by indomethacin and ethanol, presenting an effect comparable to Omeprazole and ranitidine [9]. Moreover, the methanolic extract of *C. pentandra* roots showed antiulcerogenic activity in pylorus ligation and ethanol-induced ulcers [17]. These previous results validate the traditional use of *C. pentandra* in the treatment of stomach pain and ulcer. *Ceiba speciosa* is a tropical tree and it is a native species of the Atlantic Forest, with their extracts reported to have anti-inflammatory, antipyretic, and antimicrobial activities [18,19]. In the Northwest region of the state of Rio Grande do Sul (Brazil), this species is traditionally used to reduce blood cholesterol, triglyceride, and glucose levels; however, there are no scientific reports to confirm these activities [20,21]. Aqueous stem bark extract of *C. speciosa* presented a large amount of phenolic compounds and antioxidant activity, and was able to protect against ROS-induced cell death [14]. In the region of Vale do Taquari (RS), this species is widely used to treat different gastric disorders by the local community. Therefore, the aim of the present study was to evaluate the antiulcer and anti-inflammatory activities of the ethanolic extract of *C. speciosa* using complementary in vitro and in vivo approaches.

## 2. Results

### 2.1. Phytoconstituents of C. speciosa Ethanolic Extract

The ethanolic extract of *C. speciosa* stem bark was screened for the presence or absence of tannins, alkaloids, flavonoids, coumarins, quinones, saponins, and terpenes. The results are presented in Table 1.

**Figure 1:**
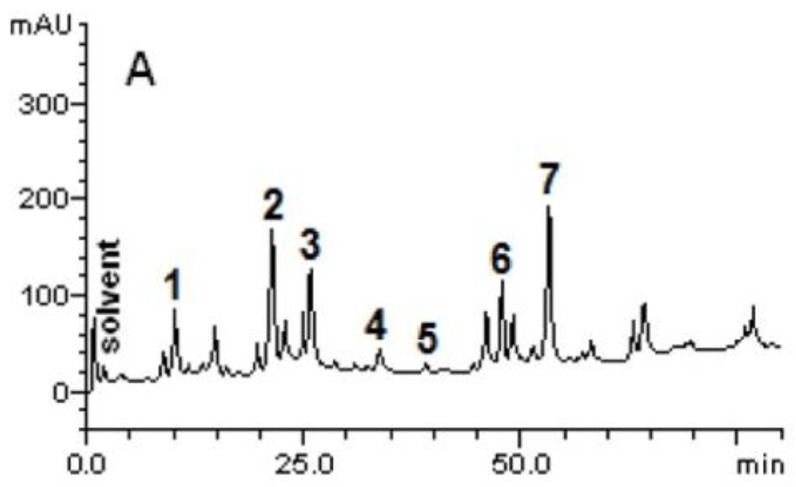
Representative profile of high-performance liquid chromatography (HPLC) of *C. speciosa* extracts. Gallic acid (peak 1), chlorogenic acid (peak 2), caffeic acid (peak 3), ellagic acid (peak 4), rutin (peak 5), quercetin (peak 6), and kaempferol (peak 7). The chromatographic conditions are described in Materials and Methods.

This preliminary qualitative screening evidenced the presence of condensed tannins, alkaloids, saponins, and flavonoids (flavonols, flavones, and flavanones) in *C. speciosa* extract. The determination of the total phenolic content (307.2 ± 6.2 mg GAE/g) by the Folin-Ciocalteau method supports the presence of phenolic compounds in *C. speciosa* ethanolic extract. Among phenolics, HPLC-DAD analysis evidenced the presence of gallic acid (Rt = 10.07 min; peak 1), chlorogenic acid (Rt = 21.45 min, peak 2), caffeic acid (Rt = 20.96 min, peak 3), ellagic acid (Rt = 34.15 min, peak 4), rutin (Rt = 38.94 min, peak 5), quercetin (Rt = 46.71 min, peak 6), and kaempferol (Rt = 58.03 min, peak 7) (Figure 1), with kaempferol and chlorogenic acid as major phenolic constituents (Table 2). In addition to phenolics, *Ceiba* genus has been reported for the presence of other classes of compounds; namely, terpenes, steroids, naphthalenes, alkaloids, glycosides, tannins, cyclopropenoid fatty acids, carbohydrates, etc. [22].

Nuclear magnetic resonance (NMR) spectroscopy can provide insights regarding the presence of a variety of organic compounds in natural matrices. This technique was used in the present work to complement HPLC analysis and to infer the presence of other structural groups than phenolics that could be present in the ethanol extract of stem bark from *C. speciosa.* Compounds were identified by comparison of 1D- and 2D-NMR spectral data with literature.

**Figure 2:**
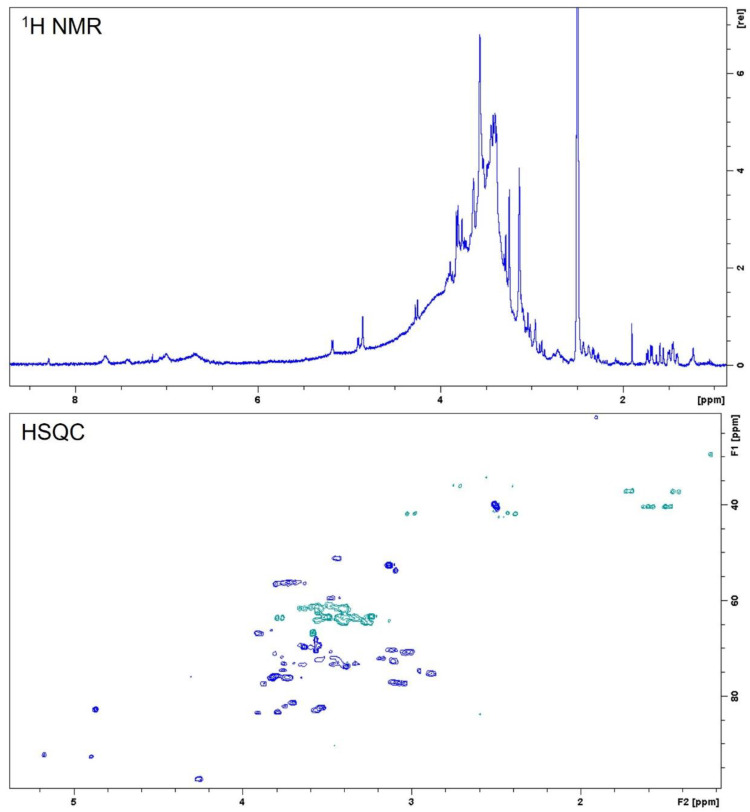
NMR (400 MHz, DMSO-d_6_) experiments (^1^H NMR and HSQC) of *C. speciosa* stem bark ethanolic extract.

Several diagnostic signals were observed in the NMR spectra of *C. speciosa* extract: Aromatic protons at δ_H_ 6.5–8.3 ppm, signals belonging to sugar components at δ_H_ 2.8–4.4 ppm that correlated with carbons at δ_C_ 60–98 ppm, and resonances in the aliphatic region between δ_H_ 1.2–3.0 ppm (Figure 2). Signals in the aromatic region can be attributed to phenolic compounds even though they appear with low intensity. On the contrary, intense peaks in the sugars’ region suggest that these molecules can appear individually or attached to different aglycone features, e.g., phenolic acids, quinic acids, flavonoids, sterols, or as components of heteropolysaccharides [22]. Furthermore, some signals observed in the aliphatic region suggest the presence of quinic acid derivatives [23,24].

### 2.2. Ceiba speciosa Antioxidant Activity

The antioxidant activity of the *C. speciosa* ethanolic extract (IC_50_ of 19.83 ± 0.34 μg/mL) was compared with the standard ascorbic acid (IC_50_ of 9.46 ± 0.36 μg/mL). The capacity of the crude extract to scavenge the DPPH radical can be considered relevant since its IC_50_ value is very close to those of the standard pure compound.

### 2.3. Cytotoxicity of C. speciosa Ethanolic Extract on Cell Lines

**Figure 3:**
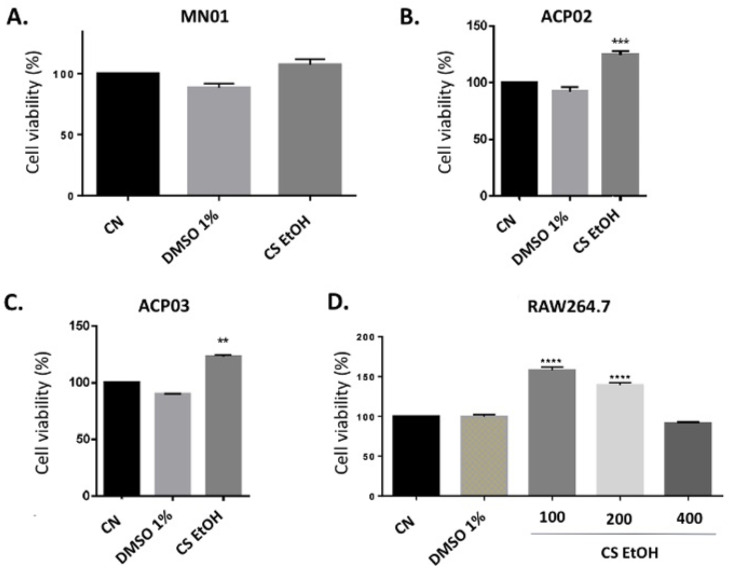
Evaluation of cell viability of MN01 (**A**), ACP02 (**B**), ACP03 (**C**), and RAW 264.7 (**D**), after 24 h of treatment with the ethanolic extract of *C. speciosa* (400 µg/mL) by the MTT method. CN—negative control, DMSO 1%—vehicle. Results are expressed as mean ± SEM (n = 3). Statistical analysis was performed using one-way ANOVA followed by Dunnett’s test. ** *p* < 0.01, *** *p* < 0.001, **** *p* < 0.0001 when compared to control.

Cell viability of ACP02, ACP03, MN01, and RAW 264.7 cell lines was assessed by the MTT method. The results are present in Figure 3A–D. In the MN01 cell line, after 24 h of treatment, the extract did not reduce the viability of the cells (Figure 3A). In the ACP02 and ACP03 cell lines, after 24 h of treatment, *C. speciosa* extract increased the cell viability in 24.8% ± 2.7% and 22.9% ± 1.7%, respectively (Figure 3B,C). The cell viability of the RAW 264.7 macrophages was not reduced when submitted to treatment with the extract at different concentrations (100, 200, and 400 μg/mL), while concentrations of 100 μg/mL (57.9% ± 4%) and 200 μg/mL (39.2% ± 3.1%) increased cell viability (Figure 3D).

### 2.4. In Vitro Inhibitory Activity of the Kinases p38α, JAK3, and JNK3

The effect of *C. speciosa* ethanolic extract was investigated on the enzymatic activity of p38α, JAK3, and JNK3. It was possible to verify (Table 3) that the extract exhibited an inhibitory effect on the three enzymes tested, with the most expressive effect on JAK3.

### 2.5. In Vitro Anti-Inflammatory Potential

The anti-inflammatory potential of the extracts was evaluated by assessing the release of the pro-inflammatory cytokine TNF-α in whole human blood and mouse macrophages. The results are present in Table 4, which indicate that the extract showed considerable inhibitory effect on whole human blood, decreasing by 50% the TNF-α release at a concentration of 100 μg/mL.

### 2.6. Ethanolic Extract of C. speciosa Decreases the Number of Leukocytes in Inflammatory Conditions

**Figure 4:**
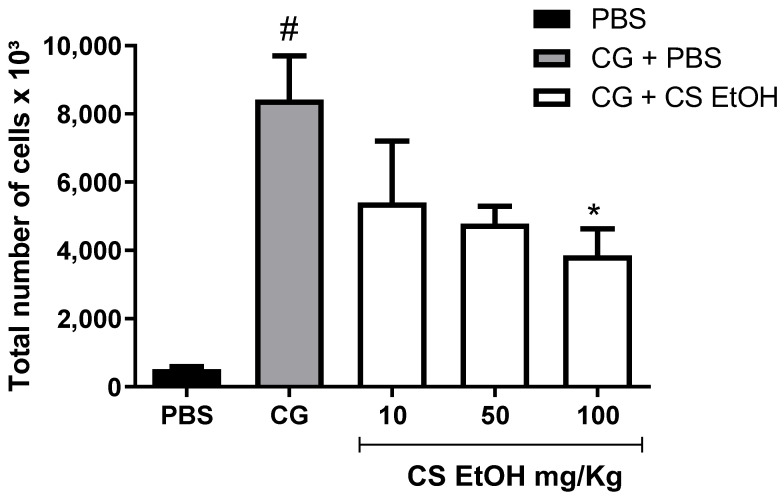
Leukocyte recruitment to the inflamed air pouch. The air pouch was inoculated with PBS (0.5 mL PBS/pouch) or carrageenan (1%). After 1 h, the extracts were applied (CS EtOH 10, 50, 100 mg/kg/0.5 mL/pouch) or PBS. The total leukocyte count was realized 24 h after treatment. The results are expressed as mean ± SEM (n = 4 to 8). The statistical analysis was realized using two-way ANOVA followed by Tukey’s post-hoc test. Significantly different *p* < 0.05 * vs. CG group and # vs. PBS group.

The in vivo effects of the ethanolic extract in the concentrations of 10, 50, and 100 mg/mL were assessed on induced inflammatory conditions by carrageenan injection into the air pouch. The first evaluation was performed by total leukocyte count from the pouch exudate (Figure 4).

The animals treated with PBS (negative control) showed a low count of leukocytes on the pouch exudate, also showing that tissue disruption to air and PBS injection did not induce inflammation. The injection of carrageenan led to a significant increase in the migration of leukocytes in the air pouch after 24 h, when compared to the control group. In the tested concentrations, a reduction in leukocyte recruitment in a dose-dependent manner in relation to the animals treated with carrageenan was observed. Mice treated with 100 mg/kg concentration of CS EtOH extracts promote significant reduction in total leukocyte of 54% after 24 h when compared to CG control group (*p* < 0.05).

### 2.7. Ethanolic Extract of C. speciosa Inhibits Protein Extravasation into Inflamed Air Pouch

**Figure 5:**
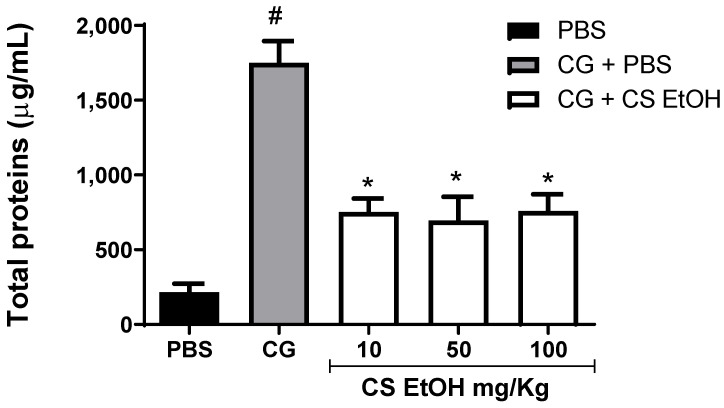
Quantification of total proteins in the air pouch. The air pouches were inoculated with PBS (0.5 mL PBS/pouch) or carrageenan 1% (CG). After 1 h, the treatment was applied (CS EtOH 10 or 50 or 100 mg/kg/0.5 mL/pouch) or PBS. The dosage of total proteins was performed after 24 h of treatment. The results were expressed as mean ± SEM (n = 4 to 8). The statistical analysis was realized using two-way ANOVA followed by Tukey’s post-hoc test. Significantly different *p* < 0.05 * vs. CG group and # vs. PBS group.

After leukocyte count, the quantification of total proteins in the air pouch as a parameter to the formation of edema was performed (Figure 5).

The highest concentration of proteins in the air pouch fluid after 24 h of treatment was observed in the carrageenan-induced animals, indicating the formation of edema, when compared to the control animals. The hydroethanolic stem bark extract of *C. speciosa* promotes a significant reduction in proteins in the extravascular space compared to the CG group in the studied concentration (Figure 5).

### 2.8. Ethanolic Extract of C. speciosa Inhibits NO Production, IL-6, and TNF-α in the Inflamed Air Pouches

**Figure 6:**
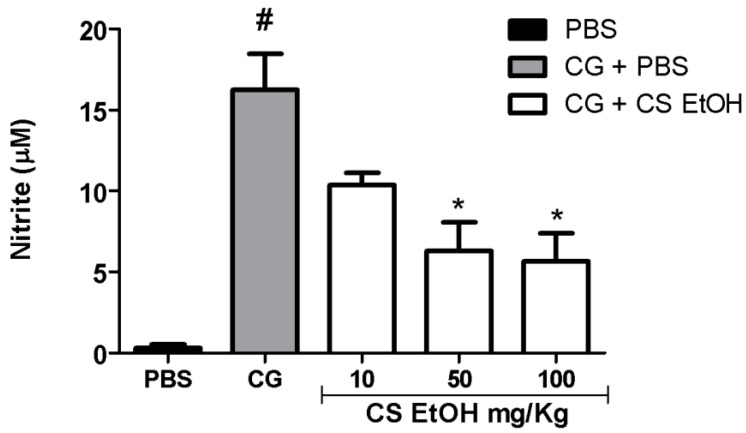
Quantification of nitric oxide (NO) in the inflamed air pouch. The air pouches were inoculated with PBS (0.5 mL PBS/pouch) or carrageenan 1% (CG). After 1 h, the treatment was applied (CS EtOH 10 or 50 or 100 mg/kg/0.5 mL/pouch) or PBS. The quantification of NO was performed after 24 h of treatment. The statistical analysis was realized using two-way ANOVA followed by Tukey’s post-hoc test. Significantly different *p* < 0.05 * vs. CG group and # vs. PBS group.

As evidenced in Figure 6, after 24 h of carrageenan injection, there was a significant increase in NO production in the air pouches, when compared to control group (PBS). The extract, in the concentrations of 100 and 50 mg/kg, was capable of significantly reverting the production of NO induced by carrageenan.

**Figure 7:**
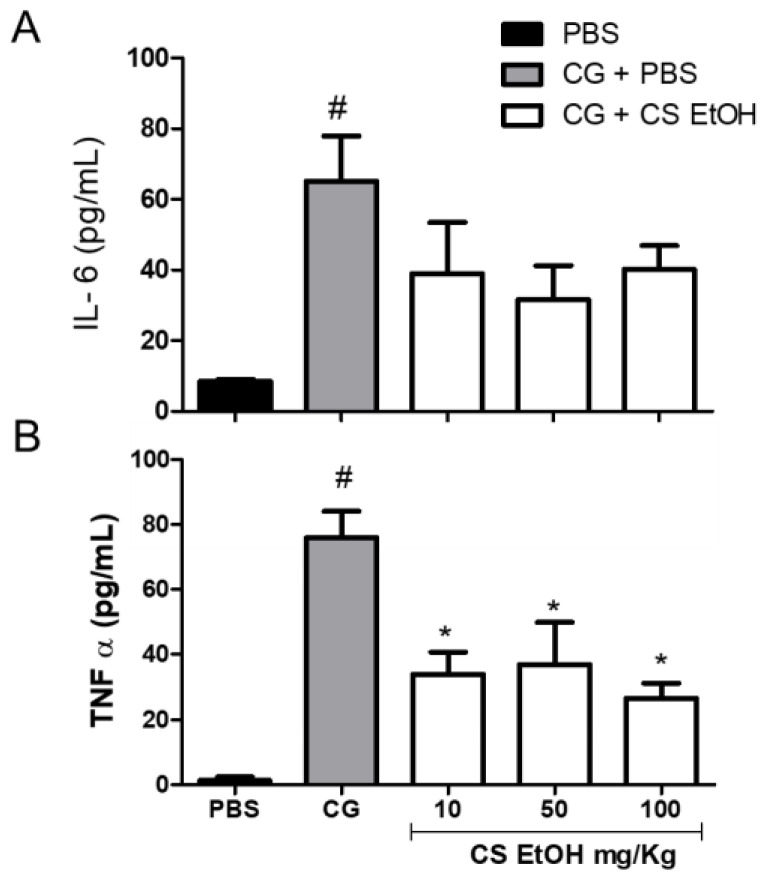
Evaluation of the release of IL-6 (**A**) and TNF-α (**B**) in the inflamed air pouch. The air pouches were inoculated with PBS (0.5 mL PBS/pouch) or carrageenan 1% (CG). After 1 h, the treatment was applied (CS EtOH 10 or 50 or 100 mg/kg/0.5 mL/pouch) or PBS. The quantification of IL-6 and TNF-α were performed after 24 h of treatment. The statistical analysis was realized using two-way ANOVA followed by Tukey’s post-hoc test. Significantly different *p* < 0.05 * vs. CG group and # vs. PBS group.

As shown in Figure 7, after 24 h, the carrageenan induced a significant production of IL-6 and TNF-α as compared to PBS. The extract, in the three tested concentrations, decreased the production of IL-6; however, no significant effects were observed (Figure 7A) when compared to the CG group. However, the hydroethanolic stem bark extract of *C. speciosa* significantly reduced the production of TNF-α at 10, 50, and 100 mg/kg compared to the CG group (*p* < 0.05) (Figure 7B).

### 2.9. Antiulcerogenic Potential of the Ethanolic Extract in Wistar Rats with Ethanol-Induced Ulcer

**Figure 8:**
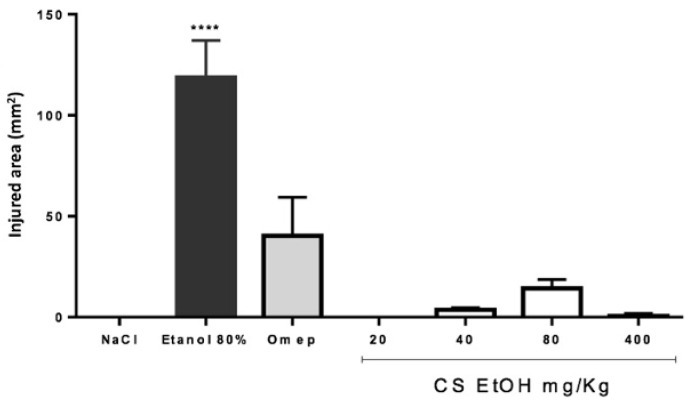
Effect of oral treatment of Omeprazole 40 mg/kg (Omep) and ethanolic extract of *C. speciosa* (CS EtOH 20, 40, 80, or 400 mg/kg) on ethanol-induced ulcer in rats. Results are expressed as mean ± SEM (n = 4 to 9). Statistical comparison was performed using one-way ANOVA followed by Dunnett’s test. **** *p* < 0.0001 when compared to NaCl (negative control, 0.9% NaCl). Ethanol 80% (control with ulcer).

The gastroprotective effect of the ethanolic extract of *C. speciosa* at different concentrations (20, 40, 80, and 400 mg/kg) was evaluated in the in vivo ethanol-induced ulcer model. The results are presented in Figure 8.

**Figure 9:**
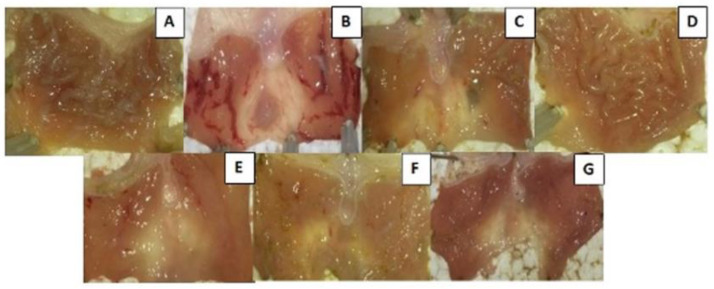
Photographs of the macroscopic appearance of the gastric mucosa of rats with ethanol-induced ulcer and pre-treated with Omeprazole or *C. speciosa* extract. (**A**) NaCl 0.9%: Control without ulcer, (**B**) ethanol 80%: Control with ulcer, (**C**) Omeprazole 40 mg/kg, (**D**) CS EtOH 400 mg/kg, (**E**) CS EtOH 80 mg/kg, (**F**) CS EtOH 40 mg/kg, (**G**) CS EtOH 20 mg/kg. The figure is illustrative and represents only one animal per experimental group.

After 1 h of treatment with different extract concentrations, animals received orally 4 mL of 80% ethanol for induction of ulcers. After 4 h, the injured area of the stomach was evaluated. Ethanol (80%) significantly induced ulcerous lesions after 4 h, in comparison to negative control, which only received saline solution (NaCl). The extract significantly prevented the formation of ulcers in all concentrations tested, did not demonstrate dose-dependent behavior, and presented greater reduction than the reference drug, Omeprazole. The macroscopic appearance of the stomach was also observed (Figure 9). The ethanol treatment has led to the formation of ulcers, also altering the coloration of the gastric mucosa, indicating a possible inflammatory process. It was observed that the gastric mucosa of animals treated with extract concentration of 400 mg/kg demonstrated a similar appearance to the animals that were not ulcer induced, with a visible rough appearance on the surface of the stomach, in addition to the presence of mucus and the non-alteration of the coloration. In the lower concentrations of the extract, and with Omeprazole, a smoother appearance of the mucosa was observed, with few ulcer lesions compared to the animals that received only 80% of ethanol. In the concentration of 20 mg/kg, there was no ulcerous lesion, but the mucosa was reddish in color.

## 3. Discussion

In the present study, the ethanolic extract from *C. speciosa* stem bark was evaluated qualitatively and quantitatively for the presence of phenolic compounds. Phenolics, mainly flavonoids, are secondary metabolites commonly found in many plant species, including species belonging to the Malvaceae family [25,26,27]. These compounds have several pharmacological effects, such as antioxidant, gastroprotective [28], anti-inflammatory, and anti-tumoral activities, and are present in most medications used in current herbal medicine [29,30].

The potential of phenolic compounds, such as chlorogenic acid, quercetin, caffeic acid, kaempferol, and gallic acid, can act by several mechanisms, including as antioxidants, in the modulation of cell signaling pathways and gene expression, turning these compounds into strong candidates for the development of new drugs [31,32,33]. Our results are in accordance with Chisom et al. (2014) [34], which found tannins, alkaloids, phenols, flavonoids, and saponins in *C. pentandra* dry powder, demonstrating a similarity between the species. Moreover, our results are similar to the ones demonstrated in Malheiros (2017), in which the *C. speciosa* extract was evaluated and 470 µg GAE/g of extract was obtained on the total phenolic content, while 307.2 GAE/g was obtained in our extract of the same plant. Both values are quite high when compared to the methanolic extract of *C. pentandra* (11.1  ±  1.63 mg GAE of extract) [26].

Primarily, we chose to perform a cell viability screening on cells of gastric origin, as well as on macrophages, given the traditional use of *C. speciosa* as anti-inflammatory and antiulcerogenic. *C. speciosa* extract showed a proliferative effect on the tested cell lines, by increasing the cell viability. Further testing is necessary to elucidate this effect, given the limitation on MTT assay, which is dependent on cell activity and presence [35].

Furthermore, studies by Kim et al. (2017) [36] and Park et al. (2012) [37] demonstrated that plants of the Malvaceae family in a similar concentration to those used in this study do not interfere with RAW 264.7 cells. Malheiros et al. (2017) [21], using *C. speciosa* ethanolic extract on human lymphocytes, demonstrated a high toxicity but with higher concentration than the ones chosen in our study. Lima et al. (2016) [38] demonstrated that the hydroalcoholic extract of *Herisantia tiubae* K. Schum. (Malvaceae) at low concentrations (6.25 to 50 μg/mL) had no toxic effect on RAW 264.7 cells; however, concentrations higher than 100 μg/mL significantly reduced the cell viability.

Other studies regarding the extract of Malvaceae family on cell toxicity demonstrated that various effects can be observed on various cell lines. *Hibiscus sabdariffa* extract presents a cytotoxic effect on AGS cells, as described by Lin et al. (2005) [39] with a greater dose than the one used in our study. In our previous studies, *C. speciosa* aqueous extract has demonstrated significant cytotoxic effect on MCF-7 breast cancer cells (Dörr et al. (2018)) [14]. In addition, Kumar et al. (2016) [40] demonstrated a similar toxicity effect of *C. pentandra* on MCF-7 cells using four different concentrations (50, 100, 250, and 500 μg/mL) of petroleum ether, ethanol, and acetone extracts.

Despite the use of different cell lines in previous studies, *C. speciosa* extracts have demonstrated low cytotoxicity. Several plants of the Malvaceae family are used for therapeutic purposes; however, there are still few studies on the effect of these species on human cells, highlighting the importance of this work.

Mitogen-activated protein kinases (MAPKs) are important proteins of cellular responses to control the environment and gene expression regulation. The p38 MAPK and JNK families are regulators of pro-inflammatory cytokines, and they are involved in many diseases, such as cancer. Inside the p38 MAPK family, the most studied and characterized protein is p38α, expressed in the major part of cells [41]. The JAK proteins belong to the tyrosine-kinase family and they are the main pathway to cytokines and growth factors signaling [42].

Therefore, MAPKs, JNK, and JAK inhibitors are known to have therapeutic potential, especially in the development of new anti-inflammatory drugs. The results of our evaluation in regard to the enzymatic activity of p38α, JAK3, and JNK3 showed that the ethanolic extract of *C. speciosa* has an inhibitory effect on these enzymes, with the most expressive effect observed on JAK3. Inhibitors of JAK3 have been pointed out as potential anti-inflammatory drugs [43]. The JNK protein is considered a potentially relevant target in the treatment of inflammatory diseases. It regulates the activity of T cells and the synthesis of pro-inflammatory cytokines, such as IL-2, IL-6, and TNF-α [44].

According to Mitsuyama et al. (2006) [45], the JNK enzyme has a role in gastric diseases, since the treatment with the inhibitor SP600125 reduced the extension of the ethanol-induced ulcers in rats. The inhibitory effect presented by the ethanolic extract may be related to the presence of phenolic compounds. Kaempferol hindered the metastasis in osteosarcoma cells by inhibition of p38 and JNK [46]. Moreover, it presented an inhibitory effect on inflammation signaling pathways by its inhibitory activity on MAPKs, including p38 and JNK [47,48]. Caffeic acid, another phenolic compound, reduced the migration of skin cancer cells by regulation of p38 [49], and quercetin decreased the production of TNF-α in obese rats, presenting an anti-inflammatory effect [50]. Both compounds were found in the ethanolic extract of *C. speciosa*, which could be related to the effects obtained in this work.

The MAPK pathway regulates the pro-inflammatory cytokines production, such as IL-6 and TNF-α. This is a proinflammatory cytokine, immediately released after an inflammatory stimulus, initiating several intracellular events, and having a fundamental role in both acute and chronic inflammations. It results in the activation of nuclear factor *kappa* B (NF-κB), as well as promoting the liberation of other pro-inflammatory cytokines, chemokines, and proteases [51].

Excessive production of TNF-α is directly associated with several inflammatory disorders, such as arthritis and inflammatory bowel disease and the regulation can be an effective therapeutic strategy in these diseases [52]. Based on the effects observed on enzymes involved in inflammatory processes, we evaluated the effect of *C. speciosa* ethanolic extracts on the release of TNF-α in different samples, in human blood, and murine macrophages. Regarding macrophages, the results show that the extract reduced the release of TNF-α, in contrast to what was observed in human whole blood. These results corroborate the importance of using different methodologies in the evaluation of biological effects.

Moreover, Fehr et al. (2015) [53] found different TNF-α values using blood samples and cells of different species. In previous studies, it was demonstrated that the inhibition of p38 by SB203580 in LPS-induced mice significantly reduced the TNF-α and IL-6 production [54]. Furthermore, the ethanolic extract evaluated in our study presented a significant inhibitory activity of TNF-α. Therefore, we may hypothesize that the anti-inflammatory effect presented by the ethanolic extract is related to its inhibitory activity on p38 MAPK pathway.

Inflammation is a complex phenomenon, which involves a highly modulated interaction by chemical mediators, leading to the migration of inflammatory cells and increased vascular permeability, causing protein exudation and edema [55,56]. The in vivo effects of *C. speciosa* ethanolic extract at concentrations of 10, 50, and 100 mg/kg were evaluated on inflammatory conditions induced by the injection of carrageenan in the air bubble. The first assessment is with the total leukocyte count of the bubble exudate. Corroborating the results obtained by Fronza et al. (2016) [57] and Marques et al. (2017) [58], we also found a significant increase in the migration of leukocytes in the air pouch after 24 h of carrageenan injection, when compared to the control group. The capacity of the extract to revert the damage caused by carrageenan was shown with the reduction in the leukocyte recruitment in a dose-dependent manner in relation to the animals treated with carrageenan.

Interestingly, the treatment with the extracts demonstrated the opposite effect when compared to the leukocyte count; namely, in the highest concentration of the extract (100 mg/kg), the concentration of proteins was also higher. However, it was noticed that the extract, in the three concentrations used, showed no differences in relation to the negative control, demonstrating that the extract reduced the edema caused by carrageenan, with one of the main symptoms observed in inflammatory processes.

Since the extract showed significant activity in the in vitro modulation of inflammatory cytokines in whole human blood, we decided to also evaluate the formation of nitric oxide (NO) in the air pouch. NO is a reactive oxygen species (ROS) known to induce tissue and vascular damage and has an important role in the pathogenesis of inflammatory processes [59]. The use of antioxidant compounds, such as phenolics can reduce the production of NO. Similarly, this effect was observed while using concentrations of 50 and 100 mg/kg of *C. speciosa* extract, which could be related to the high phenolic content of the extract. Moreover, ref. [60] demonstrated this effect using the extract of quinoa leaves.

Given the results of the modulating effect of the extract on the inhibition of TNF-α in whole human blood and in vitro inhibition of the enzymes JAK, JNK, and p38, we decided to evaluate the effect of the extract on the release of IL-6 and TNF-α on the air pouch exudate. The ethanolic extract shows an anti-inflammatory effect using this method, while using concentrations of 100 and 50 mg/kg, exhibits an inhibitory effect both in inflammation evaluation parameters, as well as inflammatory cell recruitment and edema formation.

TNF-α plays a key role in mediating inflammatory events, involved in both acute and chronic responses [61]. IL-6 produces an acute phase response, promoting the induction of intracellular signaling cascades, which give rise to the production of other inflammatory cytokines [62]. The p38 MAPK pathway directly regulates the production of inflammatory cytokines, such as IL-6 and TNF-α. Refs. [62,63] demonstrated that the use of the p38 inhibitor, SB 203580, in mice induced with LPS, led to a significant decrease in the production of TNF-α and IL-6, also increasing animals’ survival. Therefore, we can suggest that the anti-inflammatory effect exhibited by the ethanolic extract of *C. speciosa* may be related to its inhibitory effect on the MAP38 pathway.

Other plants of the Malvaceae family, such as *Hibiscus sabdariffa*, have been reported for their effects on the production of pro- and anti-inflammatory cytokines, which inhibited TNF-α and stimulated IL-10 in mice [62]. Gossypol, a polyphenolic compound extracted from the *Gossypium* genus, promoted the reduction in TNF-α and IL-6 levels in the in vitro and in vivo studies, also reducing the expression of phosphorylation of p38 MAPK and JNK [63]. However, *Althaea rosea* increased the release of TNF-α and IL-6 production and induced the expression of p38 and JNK [36], in contrast to what was observed in our study.

However, quinic acid, another compound present in this extract also showed anti-inflammatory capacity on in vivo models, and the potential to inhibit TNF-α-stimulated phosphorylation of MAP kinase and NK-κB activation on mouse vascular smooth muscle cell line (MOVAS) [64,65]. Due to the anti-inflammatory effect exhibited by *C. speciosa* ethanolic extract in the air pouch model, together with population reports about the use of this plant in the treatment of gastric disorders, the extract was also evaluated in an in vivo ethanol-induced ulcer model. Our study demonstrated that the extract prevented the formation of ulcers in all concentrations tested. Additionally, it was observed that the gastric mucosa of rats treated with the extract exhibited an aspect similar to the animals without induced ulcer, with the presence of mucus and without alteration from staining, absence of ulcerative lesions, with reddish mucosa. It is important to emphasize that the evaluation of the images is illustrative, and other studies are necessary to understand the effects of the extract on the gastric mucosa, such as tissue histology.

Several factors may influence the protection against gastric ulcers, such as mucus production and bicarbonate secretion [66], the decrease in acid production and the reduction in mucosal damage mediated by free radicals and reactive oxygen species [67]. Mitsuyama et al. (2006) [45] demonstrated the role of JNK pathway in gastric lesions treating mice with ethanol-induced ulcer with the inhibitor SP600125. The treatment significantly decreased the extension of the lesions. The ethanolic extract of *C. speciosa* presented an inhibitory effect on JNK and p38 MAPK pathways in vitro, showing the role of these pathways in the inflammatory process involved in gastric diseases.

**Figure 10:**
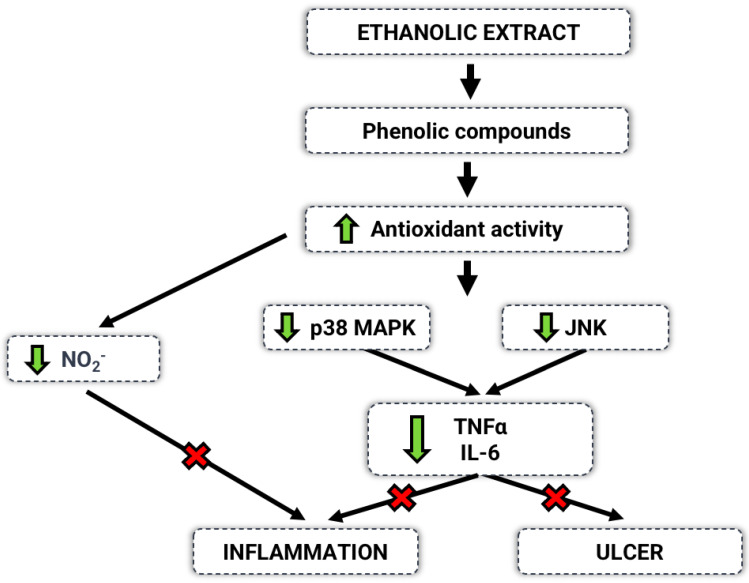
Schematic representation of the possible mechanism of action of CS EtOH extract in inflammation and ulcers. Our results demonstrate that the phenolic compounds present in the extract exhibited antioxidant potential, inhibiting oxidative stress, reducing the production of NO, and preventing the activation of p38, which, in turn, inhibited TNF−α and IL−6, exhibiting anti-inflammatory and antiulcerogenic properties.

Our findings reinforce that plants of *Ceiba* genus have potential in the treatment of gastric disorders and agree with the results obtained by Anosike et al. (2013; 2017), Bhushan et al. (2011), and Rajeswari (2013) [9,13,17,68]. We emphasize that these previous studies refer to *C. pentandra* species, with the work reported herein as the first in this field concerning *C. speciosa*. Finally, we hypothesize a possible mechanism of action of the *C. speciosa* ethanolic extract, which is demonstrated in Figure 10.

## 4. Materials and Methods

### 4.1. Chemicals

All chemicals were of analytical grade. The Dulbecco’s Modified Eagle Medium (DMEM) and RPMI-1640 culture media were purchased from Sigma^®^ (St. Louis, MO, USA) and the fetal bovine serum from Cultilab^®^ (Campinas, SP, Brazil). The reagent 3-(4,5-dimethylthiazol-2-yl)-2,5 diphenyltetrazolium bromide (MTT) was purchased from Sigma^®^ (St. Louis, MO, USA). For the TNF-α release assay, the commercial kit “Mouse TNF-α ELISA Ready-SET-Go!” (EBioscience^®^, San Diego, CA, USA) was used.

### 4.2. Plant Material and Extraction Procedure

Stem barks of *C. speciosa* were collected in Lajeado—RS, Brazil, in 2014. A voucher specimen (HVAT 516) was deposited at Herbarium of University of Taquari Valley—Univates. The stem bark was reduced to powder by knife mill and extracted by maceration with a solution of 90% ethanol (100 g of powder to 1000 mL solvent) and stored for 7 days. The solution was vacuum-filtered and evaporated in a rotary evaporator at 40 °C. The resulting dried crude extract (4.89 g) was then stored at 4 °C in an amber glass, until further chemical and biological evaluation.

### 4.3. Chemical Characterization

#### 4.3.1. Qualitative Phytochemical Screening

A preliminary and qualitative phytochemical screening was performed to evaluate the presence of flavonoids, tannins, alkaloids, coumarins, quinones, saponins, and triterpenes based upon the methodologies described by Kich et al. (2017) [69].

#### 4.3.2. High-Performance Liquid Chromatography-Diode Array Detector

High-performance liquid chromatography-diode array detection (HPLC-DAD) was performed with the HPLC system (Shimadzu, Kyoto, Japan), Prominence Auto Sampler (SIL-20A), equipped with Shimadzu LC-20AT reciprocating pumps connected to the degasser DGU 20A5, a CBM 20A integrator, a UV-Vis detector DAD (diode) SPD-M20A, and Software LC solution 1.22 SP1. Reversed phase chromatographic analyses were performed under gradient conditions using a C_18_ column (4.6 × 250 mm) packed with 5 μm particle diameter. The mobile phase was water containing 1% formic acid (A) and methanol (B), and the composition gradient was: 13% of B until 10 min and changed to obtain 15%, 30%, 50%, 60%, 70%, 20%, and 10% of B at 20, 30, 40, 50, 60, 70, and 80 min, respectively, as described by Adefegha et al. (2017) with slight modifications. Ethanolic extract of *C. speciosa* was dissolved in methanol at a concentration of 10 mg/mL. The presence of seven antioxidant compounds was investigated, namely, gallic acid, chlorogenic acid, ellagic acid, caffeic acid, quercetin, rutin, and kaempferol. Identification of these compounds was performed by comparing their retention times (Rt) and UV absorption spectra with those of commercial standards. The flow rate was 0.5 mL/min, injection volume 40 μL, and the detection wavelengths were: 257 nm for gallic acid, 325 nm for caffeic, ellagic and chlorogenic acids, and 366 nm for quercetin, rutin and kaempferol. All the samples and mobile phases were filtered through a 0.45 μm PTFE membrane filter and degassed by ultrasonic bath prior to use. Stock solutions of standard references were prepared in the HPLC mobile phase in a concentration range of 0.030–0.500 mg/mL. Peak identification was confirmed by comparing the Rt with those of reference standards and by UV-Vis spectra (200 to 600 nm). All chromatographic operations were carried out at room temperature.

#### 4.3.3. Nuclear Magnetic Resonance Analysis

For nuclear magnetic resonance (NMR) analysis, *C. speciosa* extract (c.a 20 mg) was dissolved in 0.5 mL of dimethyl sulfoxide (DMSO-*d6*) (Sigma-Aldrich, St. Louis, MO, USA). NMR spectra were acquired on a Bruker 400 apparatus operating at 400.13 MHz for ^1^H and at 100.12 MHz for ^13^C. Chemical shifts (*δ*) are expressed in ppm and referenced to the residual solvent signal (^1^H = 2.50 ppm and ^13^C = 39.50 ppm).

### 4.4. Antioxidant Capacity of Ceiba speciosa Extract

#### 4.4.1. Determination of Total Phenolic Content

The determination of total phenolic content (TPC) of the plant extract was evaluated by the Folin-Ciocalteau method as described by Singleton and Rossi (1965, adapted) [70]. The content of total phenolics was expressed as milligrams of gallic acid equivalents (GAE) per gram of extract (mg GAE/g).

#### 4.4.2. Diphenyl-2-picrylhydrazyl (DPPH) Radical Scavenging Activity

The 1,1-diphenyl-2-picrylhydrazyl (DPPH) radical scavenging activity was performed according to Mensor et al. (2001) with some modifications. An aliquot of 10 µL of *C. speciosa* extract of different concentrations was added to 990 µL of DPPH ethanol solution (0.1 mM) to obtain final concentrations of 3.12–100 µg/mL and the mixtures were then incubated in the dark for 30 min at room temperature. Ascorbic acid (AA) was used as standard. The ability of samples to scavenge the DPPH radical was calculated using the following equation:

$$\mathrm{AA}\%=\frac{ABS\;\mathrm{DPPH}-ABS\;sample\;}{ABS\;\mathrm{DPPH}}\times 100$$

Results are expressed as mean values ± SEM (standard error of the mean). IC_50_ values (µg/mL) were also determined for the extracts with highest activity (DPPH reduction > 50%).

### 4.5. In Vitro Cell Assays

#### 4.5.1. Cell Lines and Culture Conditions

The cells MN01 (gastric epithelium) and human gastric adenocarcinoma (ACP02—cardia region and ACP03—pyloric antrum region) cells were donated by Dr. Diego Bonatto (Universidade Federal do Rio Grande do Sul—UFRGS) and maintained in RPMI 1640 medium supplemented with 10% fetal bovine serum (FBS) antibiotic/antimycotic solution. Murine macrophages (RAW 264.7) cells were purchased from BCRJ bank (Cell Bank of Rio de Janeiro). Cells were cultured with DMEM (Dulbecco Modified Eagle Medium) medium and supplemented with 10% FBS and 1% antibiotic/antimycotic solution. The cells were incubated at 37 °C and 5% CO_2_ in a humidified atmosphere.

#### 4.5.2. Cell Viability Assessment

The assessment of cell viability was performed according to the MTT colorimetric assay [59]. RAW 264.7 cells were plated at a density of 3 × 10^3^ cells/well, while MN01, ACP02, and ACP03 were plated at a density of 2 × 10^3^ cells/well in a 96-well plate. Cells were treated with 400, 200, and 100 μg/mL of *C. speciosa* ethanolic extract for 24 h. After 3 h of incubation with MTT, the absorbance was read at 570 nm using an ELISA microplate reader (ThermoFisher Scientific, Vienna, Austria). Results are expressed as percentage of control.

#### 4.5.3. Human Peripheral Blood Mononuclear Cells (PBMC) Isolation

Peripheral blood mononuclear cells were obtained from healthy volunteers after they were informed, and a consent was obtained. This study was approved by the Ethics Research Committee of Univates. Isolation of PBMCs was performed by Ficoll gradient centrifugation, as previously described [28].

#### 4.5.4. Anti-Inflammatory Potential

The anti-inflammatory potential of the extract was evaluated by assessing the release of the pro-inflammatory cytokine TNF-α in whole human blood, and was performed as described by Bauer et al. (2016) [71]. Whole blood was treated with *C. speciosa* extract at concentrations of 100, 50, 25, and 12.5 μg/mL for 15 min, and thereafter stimulated by lipopolysaccharide (LPS) (1 µg/mL) for 4 h. The supernatant was collected and the plasmatic concentration of TNF-α was assessed by the ELISA method. The results are presented as percent of inhibition.

#### 4.5.5. Cell-Free Kinase Assay

*Ceiba speciosa* ethanolic extract was screened for p38 mitogen-activated protein kinase (MAPK), c-Jun N-terminal kinase 3 (JNK3), and Janus kinase 3 (JAK3) inhibition. The inhibitory potential was assessed by previously established ELISA assays measuring the inhibition of p38α- and JNK3-mediated ATF-2 phosphorylation, and JAK3-mediated ATP phosphorylation [72,73]. The half maximal inhibitory concentration (IC_50_) of the extract was calculated.

### 4.6. In Vivo Experiments

#### 4.6.1. Animals

All procedures with animals were carried out according to the National Institutes of Health (NIH) Guide for the Care and Use of Laboratory Animals. All animals were maintained in an appropriate shelter, with water and food *ad libitum*. Wistar rats were used in the experiments of ethanol-induced ulcer and *Swiss Mus musculus* mice were used in the air pouch assay. The research was approved by the CEUA (Committee on Ethics in the Use of Animals) of the Universidade de Vila Velha (UVV) under Protocol number738-2017 and Universidade do Vale do Taquari (Univates), under Protocol number 001/2015.

#### 4.6.2. Air Pouch

In vivo anti-inflammatory activity of *C. speciosa* ethanolic extract was investigated in the mice carrageenan-induced inflammation air pouch model, as previously described [57,58]. For this experiment, *Swiss Mus musculus* mice were used (25–35 g). Initially, the animals were anesthetized with a ketamine (80 mg/kg) and xylazine (15 mg/kg) solution and the animals’ back was shaved; then, 3 mL of sterile air was injected subcutaneously into the midline of the dorsal region with a needle through a sterile filter. On the fourth day, 2 mL of sterile air was injected into the preexisting air pouch. After 2 days, on the sixth day, the animals were randomly divided into 5 groups with 6 or 8 mice. One of the groups received 0.5 mL of phosphate-buffered saline (PBS) and the rest of the groups received 0.5 mL of carrageenan 1%, directly into the air pouch. After 1 h, controls (only PBS and PBS + carrageenan) received PBS (0.5 mL/pouch) and the other 3 groups were treated with 100, 50, and 10 mg/kg of plant extract (0.5 mL/pouch). After 24 h, all animals were euthanized with an overdose of anesthetics, ketamine, and xylazine.

#### 4.6.3. Evaluation of Leukocyte Accumulation into the Air Pouch

After euthanizing the animals, 2 mL of sterile PBS containing 3.6% of sodium citrate was injected into the air pouch and gently massaged for 1 min. The air pouch was opened, and the exudates were collected and stored in ice. The cells were counted using Turk’s solution in a Neubauer chamber. The exudate was centrifuged at 400 rpm for 10 min at 4 °C, and the supernatant was used to evaluate proteins and cytokines.

#### 4.6.4. Total Protein Quantification

Total proteins were quantified in the supernatant obtained from the air pouch, according to the method described by Bradford (1976) [74]. The experiments were performed in a 96-well plate, by adding 5 µL per well of sample and 250 µL of Coomassie Brilliant Blue. Measures were performed in an ELISA microplate reader at 595 nm and the results were expressed in µg/mL.

#### 4.6.5. Cytokines and Lipid Mediators’ Measurement

The obtained cell-free fluid was used to measure nitric oxide (NO), IL-6, and TNF-α levels. The dosage of NO was evaluated by the Griess method (Green et al. (1982). The experiments were performed in a 96-well plate and the measures were performed in an ELISA microplate reader at 540 nm. Results were expressed in µM of NO. The quantification of TNF-α and IL-6 was performed according to the manufacturer’s instructions for each kit and measured at 450 nm in an ELISA microplate reader. The concentration of cytokines was expressed in pg/mL.

#### 4.6.6. Antiulcerogenic Effect on a Model of Ethanol-Induced Ulcer in Wistar Rats

The antiulcerogenic activity of *C. speciosa* extract was evaluated by an experimental model of gastritis, as described by Haule et al. (2012) [28]. For the experiments, *Wistar* rats (150–380 g) were used. The rats were divided into 7 groups of 6 or 9 rats. Two groups were treated with 1 mL of saline orally administered (negative control without ulcer and negative control with ulcer); one group was treated with Omeprazole (40 mg/kg), a standard antiulcer, and the other groups were treated with 1 mL of plant extract (400, 80, 40, and 20 mg/kg). After 1 h, 4 mL/kg of 80% ethanol were orally administered to the rats (except for the group of negative control without ulcer) for induction of the ulcer lesions. After 4 h, all animals were sacrificed by thiopental sodic (80 mg/kg) and the stomach was removed and opened along the greater curvature and photographed. The ulcer lesion was measured with *ImageJ 1.50i* software to determine the size of the lesioned area in mm^2^.

### 4.7. Statistical Analysis

Statistical analyses were performed using GraphPad Prism 6.0 (GraphPad Software, Inc, San Diego, CA, USA). All data were expressed as mean ± SEM. Statistical significance was evaluated using ANOVA followed by Dunnett’s test. A *p*-value ≤ 0.05 was considered as statistically significant.

## 5. Conclusions

In this study, it was possible to expand the knowledge in regard to the bioactive effects of *C. speciosa*, highlighting the importance of ethnopharmacological reports in plants’ prospection with therapeutic potential.

The ethanolic extract of *C. speciosa* stem bark was subjected to a series of in vitro assays, evidencing no cytotoxicity and significant effects on enzymes involved in several inflammatory processes, as well as demonstrating therapeutic potential for the treatment of gastric diseases. Additionally, the performed studies on in vivo models evidence that the gastric mucosa of rats treated with the extract demonstrated an aspect similar to the animals without ethanol-induced ulcer, revealing that *C. speciosa* extract can be promising in gastrointestinal therapeutics.

In addition to more in vivo toxicity evaluations, further studies are needed to understand the mechanisms of action of *C. speciosa* compounds, aiming at the development of novel multitarget drugs with gastroprotective and anti-inflammatory properties to overcome undesirable side effects of several drugs used in current therapy of gastrointestinal impairments.

## Figures and Tables

**Table 1:** Phytochemical characterization of *Ceiba speciosa* extract.

Secundary Metabolites		CS EtOH
Tannins	Hydrolysable	−
	Condensed	+
Alkaloids		+
Flavonoids	Flavonols	+
	Flavones	+
	Flavonones	+
Coumarins		−
Quinones		−
Saponins		+
Steroids/Triterpenes		−

(+) Presence; (−) Absence.

**Table 2:** *Ceiba speciosa* ethanolic extract composition.

Compounds	CS EtOH (mg/g)
Gallic acid	1.98 ± 0.02 ^d^
Chlorogenic acid	4.15 ± 0.01 ^b^
Caffeic acid	3.76 ± 0.04 ^c^
Ellagic acid	0.59 ± 0.01 ^e^
Rutin	0.17 ± 0.01 ^f^
Quercetin	2.03 ± 0.03 ^d^
Kaempferol	5.11 ± 0.05 ^a^

Results are expressed as mean ± standard deviations (SD) of three determinations. The averages followed by different letters differ by the Tukey test at *p* < 0.01.

**Table 3:** Inhibition potential (IC_50_) of the *Ceiba speciosa* ethanolic extract on enzymatic activity (n = 3).

Enzyme	CS EtOH	Standard
p38-α	1.66 ± 0.24 µg/mL	0.052 ± 0.02 µM (SB 203580)
JNK3	5.40 ± 0.21 µg/mL	0.164 ± 0.06 µM (SP 600125)
JAK3	8.34 ± 0.83 ng/mL	2.603 ± 0.05 nM (CP 690550)

**Table 4:** Inhibitory potential of *Ceiba speciosa* extract on TNF release on whole human blood.

Concentration of the Extract (µg/mL)	CS EtOH % Inibition
SB203580	88.3 ± 6.3
100	50.3 ± 10
50	33.3 ± 9
25	15.2 ± 0.5
12.5	13.8 ± 5

Percentage of inhibition at 100 µM (except or SB203580, 10 µM). Mean ± SD are shown (n = 2). Basal (negative control): 0 pg/mL, Positive controls (LPS without test substance): 359.3 ± 121.14 pg/mL.
